# A THz graphene-on-hBN stack patch antenna for future 6G communications

**DOI:** 10.1038/s41598-025-16695-x

**Published:** 2025-09-23

**Authors:** Elana P. de Santana, Kun-Ta Wang, Sergi Abadal, Daniel Stock, Zhenxing Wang, Anna Katharina Wigger, Eduard Alarcón, Max C. Lemme, Peter Haring Bolívar

**Affiliations:** 1 Institute of High Frequency and Quantum Electronics, University of Siegen, 57076 Siegen, Germany; 2 AMO GmbH, Advanced Microelectronic Center Aachen (AMICA), 52074 Aachen, Germany; 3 Chair of Electronic Devices, RWTH Aachen University, 52074 Aachen, Germany; 4 NanoNetworking Center in Catalunya (N3Cat), Universitat Politècnica de Catalunya, 08034 Barcelona, Spain

**Keywords:** Electrical and electronic engineering, Electronic properties and devices, Electronic properties and devices

## Abstract

Wireless communications have progressively tapped into higher frequency bands seeking higher bandwidth and integration, opening the door to short-range applications such as data kiosks, wireless chip interconnects, or intra-body networks. Graphene antennas working at terahertz (THz) frequencies are theoretically smaller in size compared to metallic antennas working at the same frequency, pushing the boundaries of integration further. However, such miniaturization ability has not yet been experimentally validated. This study presents the first working THz antenna based on chemical vapor deposited (CVD) monolayer graphene. The antenna, placed on the hexagonal Boron Nitride (hBN) buffer layer, comprises a multi-layer stack of two graphene patches separated by a thin dielectric, resulting in a significantly more efficient antenna than a standard one-layer graphene antenna. The proposed antenna shows a resonance frequency of 250.7 GHz and a gain of -9.5 dB. The miniaturization and frequency tuning capabilities of graphene antennas make the proposed graphene stack patch antenna a valuable asset for 6G short-range communications. Additionally, the proposed graphene stack antenna can be integrated in the back-end-of-line with CMOS manufacturing techniques and applied to future THz communication systems.

## Introduction

The millimeter-wave (mm-wave) and terahertz (THz) frequency bands are among the key technologies being investigated for future 6G communication systems. The low latency and larger bandwidth provided by these frequency ranges will eventually lead to Tbps rates tackling the higher demands for data transfer of innovative technologies, such as autonomous vehicles, virtual and augmented reality, industry 4.0, and others^1–4^. Moreover, due to the reduced size of the passive devices at higher frequencies, more antennas can be packed in the same area leading to arrays with increasingly directional beams.

Due to high spreading losses and molecular absorption in this frequency range, THz communications find relevant applications in short-range cases where area constraints dominate, or extreme bandwidth is required. As illustrated recently, THz and mm-wave intra-chip and chip-to-chip wireless communications can complement the already limited wired interconnects within computing packages^5^ to relieve communication bottlenecks of future computing platforms with hundreds or thousands of processors within one package. The implementation of miniaturized antennas and transceivers as wireless interconnects within computing packages will lead to low-latency reconfigurable communication links, alleviating the dense wired interconnect fabric^5^.

Technological advances at the antenna side of communication systems are required for the ubiquitous implementation of future 6G systems at the aforementioned frequency bands. Graphene-based antennas are innovative components that could complement standard metallic antennas due to the long-standing predictions for significantly reduced dimensions and frequency tunability capabilities^5–8^ at the THz range. Graphene is a carbon-based 2D material widely studied by many research groups due to its unique mechanical, optical, thermal, and electrical characteristics. Its distinct energy band dispersion relation permits the graphene conductivity to be modified significantly chemically, by doping, or electrically, by an applied bias voltage. Due to this, graphene has been used in various electronic applications^10–14,14,15,15,16,16–21^. The possibility of tuning the graphene conductivity is especially appealing at THz frequencies due to the excitability of strongly confined surface plasmon polaritons (SPP) at a graphene/dielectric interface at this frequency range^22^. These electromagnetic (EM) surface waves wavelengths are dependent on the graphene conductivity, which leads to the possibility of frequency-tunable graphene THz antennas. Additionally, the SPP wavelength is smaller than in free-space, making graphene THz antennas significantly smaller than standard metallic antennas. Graphene antennas could therefore lead to cost-effective 6G components, by the potential reduction of antenna area, and the compatibility to flexible substrates^23^. Furthermore, more agile and flexible communication protocols and systems could be realized using the tunability potential of graphene antennas^24^. However, such antennas have not been experimentally demonstrated until now.

Plasmonic graphene THz antennas working at the low range of the THz spectrum have a huge potential, therefore. However, since their original inception, in the year 2012^25^, until now, they have only been studied theoretically and by simulations^3,26–38^. Most of the published work regarding graphene antennas is based on simple monolayer graphene patches without any biasing possibility. Similar graphene stack antennas to the one proposed here have been presented^39–43^ employing simulation only. These antennas, however, are based on dipoles with photomixers as a THz source that cannot easily be integrated into classical transceivers, as typical highly doped substrates would degrade dipole emission significantly. In the works of Hossenininejad et al. (2017)^44^ and Abadal et al. (2017)^6^, a photoconductive dipole graphene stack antenna is analyzed based on a dielectric spacer between the graphene sheets with high and low dielectric constant values at the frequency range of 2 - 3 THz. In 2018 another dipole photoconductive graphene antenna consisting of a stack of graphene/polymethyl methacrylate (PMMA)/graphene was analyzed in the frequency range of 1 - 5 THz^42^. The above-mentioned papers are based on simulations with very high graphene conductivity values, lacking experimental proof of the realization of working antennas.

Indeed, as further theoretical analysis showed^45^, competitive graphene antenna characteristics and efficiencies, in comparison to its classical metallic counterpart, put very stringent requirements on a very high mobility (i.e. low scattering rate) and at the same time on a high carrier density (i.e. a high Fermi level) of the graphene antenna material. The excellent potential illustrated by most theoretical predictions to date stems from idealized graphene structures using material properties close to the ideal optimum of exfoliated graphene flakes with record mobilities and at the same time high carrier densities. In reality, graphene compromises mobility when scalable graphene fabrication routes like Chemical Vapor Deposition (CVD) are used, and compromises mobility further, when higher carrier densities are used. This is the deeper reason why despite very promising graphene antenna theoretical predictions, experimental demonstration has remained elusive for more than a decade. Such an experimental demonstration is fundamentally difficult and has not yet been successful, as realistically integratable graphene material mobilities and Fermi levels come at the expense of a significantly degraded antenna efficiency compared to a classical metallic antenna^3^.

Only a few papers published can be found with experimental results of graphene antennas^13,30,46–53^. But these are experiments performed at very low frequencies in the 1–12 GHz frequency range^13,47–49,51,52^, where graphene does not present plasmonic behavior and, consequently, no fundamental advantages in terms of antenna size in comparison to classical metallic antennas are achievable. Other papers with graphene antenna demonstration in a higher frequency range were not made with monolayer graphene - being these antennas, in fact, metal-based^46^ or carbon-based antennas with the antenna elements having more than 10 graphene layers^30,50^ and, consequently, rather the performance of standard graphite.

In this paper, we demonstrate, for the first time, emission coming from a graphene-based antenna at terahertz frequencies. The antenna consists of a CVD monolayer graphene stack being fed electronically by a signal line at the frequency range of 220–325 GHz. The stack comprises a bottom graphene patch covered by an insulating dielectric layer and a top graphene patch. The graphene stack proposed here leads to significantly relaxed requirements for the material parameters and is therefore used to increase the overall efficiency and gain of the antenna compared to a simple monolayer graphene antenna type. This allows us to demonstrate graphene antenna emission experimentally for the first time. Since the antenna in this configuration is all-electronic based and is embedded on top of a metallic plane and polyimide (PI) layer, it permits a simple route for future integration within transceivers for future THz communications as a back-end-of-the-line (BEOL) process. The antenna can be manufactured above the transceiver semiconductor circuit and fed through substrate vias. Besides, it provides a simple geometry to apply a significant electrostatic bias to the graphene antenna. Moreover, the results support the miniaturization claims made in past simulation works, as a 10% reduction in the resonance frequency over a same-size metallic counterpart is achieved.

In the subsequent sections, we present the design, simulation, and measurement results of the proposed antenna. The antenna fabrication is explained, as well as the antenna measurement setup. Finally, the far-field antenna measurement results are presented and discussed.

## Results

### Antenna design and simulation

**Fig. 1:**
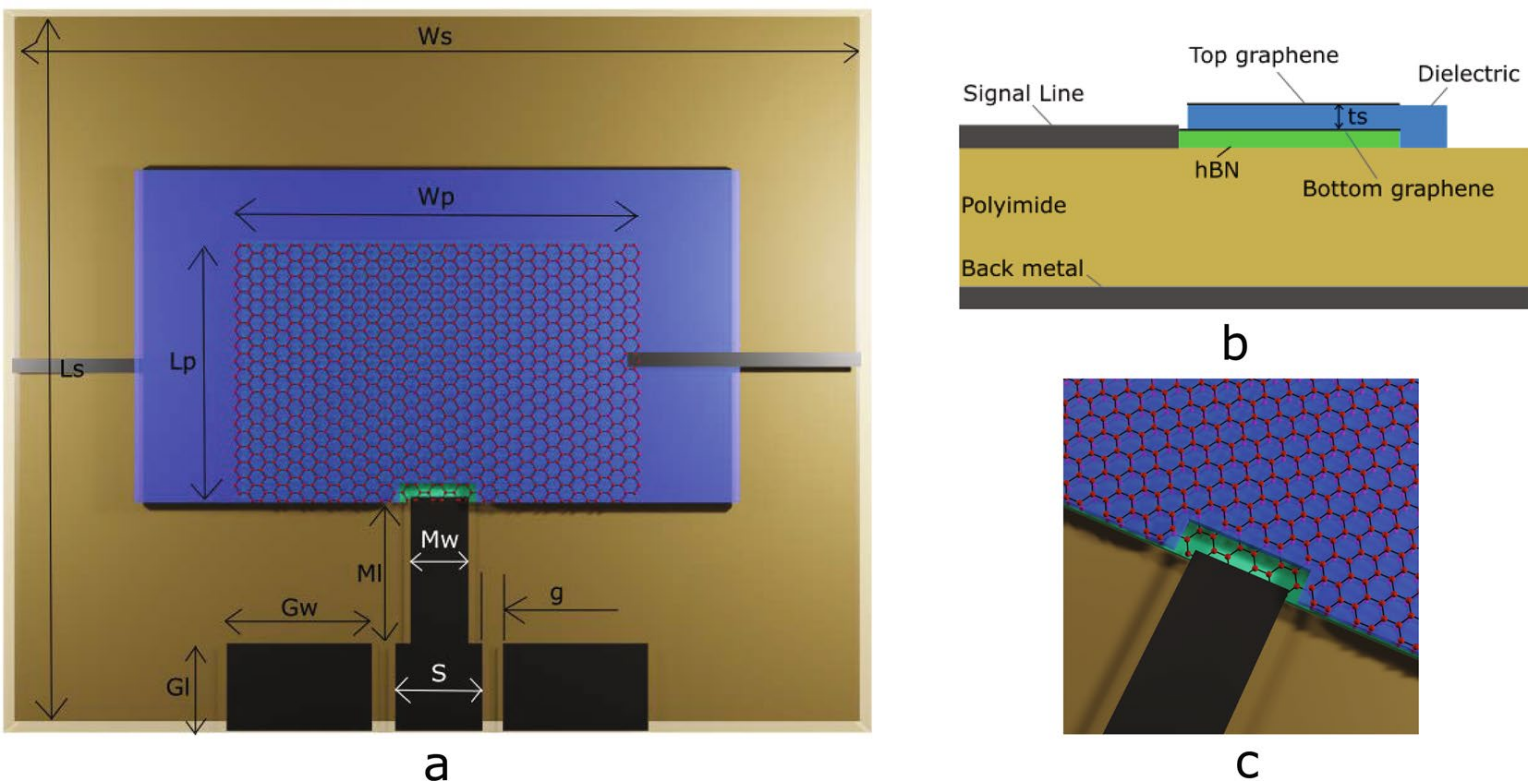
Graphene patch stack antenna design with a resonance frequency of f_r_ = 280 GHz with polyimide substrate, GSG pads, a hBN layer (green area), and a dielectric layer (blue area) between the graphene patches on hBN. (**a**) Antenna top view showing the radiating stack and the left and right graphene biasing lines. (**b**) Antenna cross-section along the central line showing the proposed stack. A hBN layer exists between the polyimide substrate and the bottom graphene. (**c**) Detailed illustration of the connection of the signal line with the bottom graphene patch (the cut-out prevents contacting the top graphene layer). Dimensions in figures are not to scale.

The proposed graphene stack patch antenna is shown in Fig. 1. It consists of a resonant patch on top of a polyimide ($\epsilon$_r_ = 3.5) substrate of 50 $\mu$m thickness with a back-metal plane. The resonant patch element is fed by a coplanar waveguide (CPW) designed to match a ground-signal-ground (GSG) probe of 50 $\Omega$ impedance. The proposed resonant element is a stack of graphene/Al_2_O_3_/graphene. The Al_2_O_3_ (alumina) thickness of 80 nm permits the tuning of bottom/top graphene by applying an electrostatic bias between the two graphene sheets. The signal line contacts the lower graphene patch within the resonant stack. To avoid the contact of the signal line with the top graphene patch (which would short-circuit the biasing possibility between the two graphene patches of the stack), a cut-out of 5 $\mu$m distance from the signal line is used. Both the bottom and the top graphene layers of the stack are contacted laterally by additional biasing lines, illustrated in Fig.1 by the lines on the left (bottom graphene) and right (top graphene).

The antenna stack is placed on CVD hBN to protect graphene from unwanted doping from the substrate. Since hBN possesses only a 1.8% lattice mismatch with graphene^54^, it significantly reduces the scattering centers at the interface to graphene^54^. Using hBN as a buffer layer, instead of directly transferring graphene on the polyimide substrate, improves the electrical properties of graphene. This can be proven by resistance measurements between two terminals of the graphene patch.

The dimensions of the radiating patch were calculated using standard antenna design equations^55^ and adjusting all dimensions with full 3D EM simulations. The antenna was designed to resonate at 280 GHz, leading to the dimensions described in Table 1.

**Table 1:** Design parameters of the proposed graphene stack antenna.

Parameter	Value ($\upmu$m)
Patch width (W_p_)	355
Patch length (L_p_)	260
Substrate width (W_s_)	710
Substrate length (L_s_)	570
Signal pad width (S)	70
Ground pad width (G_w_)	100
Ground pad length (G_l_)	60
Match line width (M_w_)	40
Match line length (M_l_)	190
Gap (g)	10
Dielectric thickness (t_s_)	0.08
Substrate thickness (t)	50

The surface conductivity ($\sigma$) of graphene including the intra-band and inter-band contributions is calculated using the standard Kubo formula^56^ (complete derivation in Supplementary Information), which is commonly used to model a graphene sheet^57^ and it is expressed as

1 $$\begin{aligned} \begin{aligned} \sigma (\omega )=&\frac{-ie^{2}(\omega +i\tau ^{-1})}{\pi \hslash ^{2}}\Big [\frac{1}{(\omega +i\tau ^{-1})^{2}}\int _{0}^{\infty }\Big (\frac{\partial f_{d}(E)}{dE}-\frac{\partial f_{d}(-E)}{dE}\Big )E dE-\int _{0}^{\infty }\frac{f_{d}(-E)-f_{d}(E)}{(\omega +i\tau ^{-1})^2-4(E /\hslash )^2}dE\Big ] \end{aligned}\end{aligned}$$

where $\omega$ is the radian frequency, $\mu _{\textrm{c}}$ is the graphene chemical potential, $\tau$ is the relaxation time of the excited charge carriers in the graphene, *T* is temperature, *e* is the electric charge, $\hslash$ is the reduced Planck’s constant, E = $\hslash \omega$, and $f_{\textrm{d}}$ is the Fermi-Dirac distribution described as

2 $$\begin{aligned} \begin{aligned} f_{d}(E)=(e^{(E-\mu _{c})/k_{B}T}+1)^{-1}, \end{aligned}\end{aligned}$$

where $k_{\textrm{B}}$ is the Boltzmann constant.

From Eq. (1), the intraband transitions contribution to the graphene conductivity is described as

3 $$\begin{aligned} \begin{aligned} \sigma _{intra}(\omega )=\frac{2k_{B}Te^2}{\pi \hslash ^2}ln\Big [2cosh\Big (\frac{\mu _{c}}{2k_{B}T}\Big )\Big ]\frac{i}{\omega +\frac{i}{\tau }}, \end{aligned}\end{aligned}$$

and the interband transitions contribution of graphene is expressed as

4 $$\begin{aligned} \begin{aligned} \sigma _{inter}(\omega )=\frac{ie^2}{4\pi \hslash }ln\Bigg [\frac{2\mu _{c}+\Big (\omega +\frac{i}{\tau }\Big )\hslash}{2\mu _{c}-\Big (\omega +\frac{i}{\tau }\Big )\hslash}\Bigg ]. \end{aligned}\end{aligned}$$

In the THz frequency range, the contribution of interband transitions to graphene’s conductivity is negligible. Therefore, the conductivity of graphene can be calculated directly using Eq. 3. The two main parameters that determine graphene’s conductivity are the chemical potential, which is related to the doping level in graphene, and the relaxation time of the charge carriers, which is associated with the quality of the graphene. Higher values of both the chemical potential and relaxation time will result in graphene exhibiting higher conductivity.

**Fig. 2:**
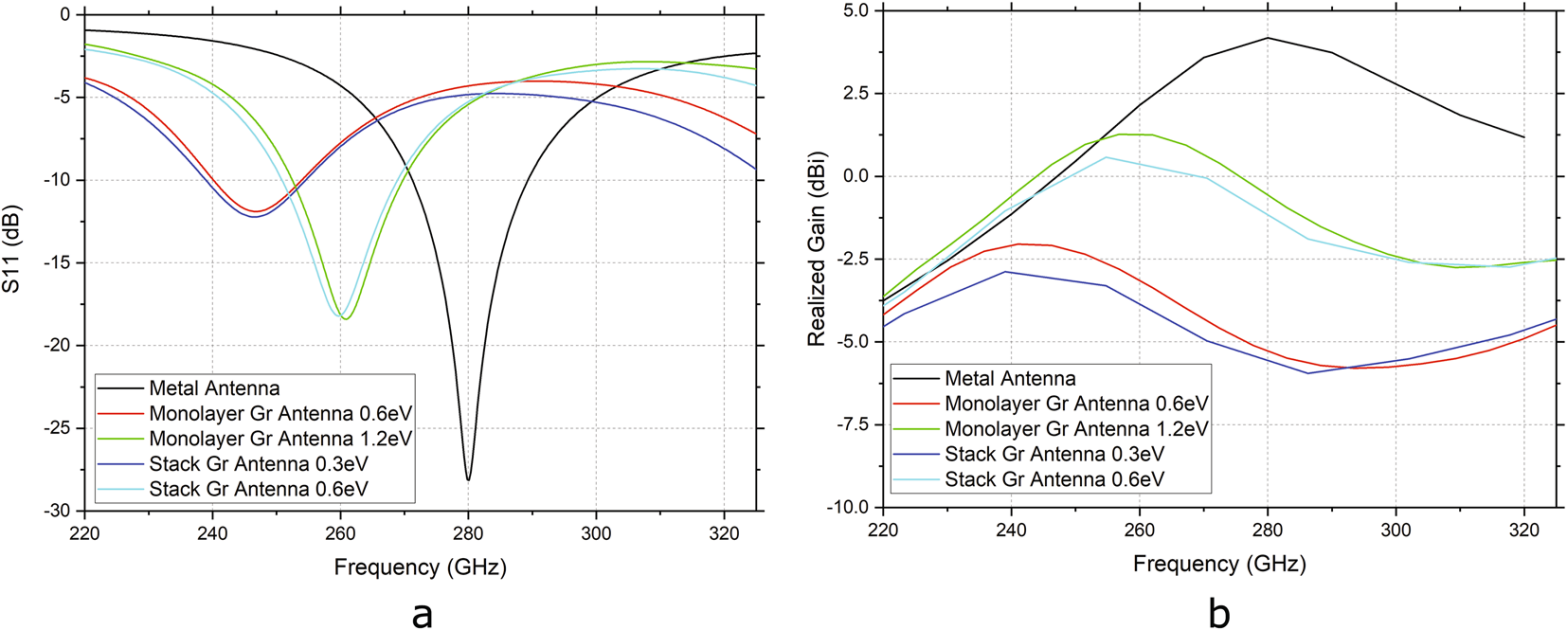
(**a**) Simulation comparison of the graphene antennas S11 with the S11 of a metallic antenna patch consisting of palladium with a thickness of 100 nm. The graphene antennas compared are a standard monolayer graphene antenna and the proposed stack antenna with graphene characteristics of 0.3 eV, 0.6 eV, 1.2 eV of chemical potential and 1.2 ps of relaxation time. (**b**) Simulation comparison of the graphene antennas realized gain at 0° with the gain of the metallic antenna patch consisting of 100 nm palladium.

The proposed graphene stack antenna is simulated with HFSS. For a better understanding of the graphene stack antenna behavior, its S11 and realized gain are compared to that of a metal antenna and a standard monolayer graphene antenna of the same dimensions. Graphene is simulated as a 2D sheet with impedance boundary condition defined by the impedance values calculated using Eq. 3 (more details in the Supplementary Information). Graphene with 1.2 ps of relaxation time and different values for the chemical potential is simulated and the results are plotted in Fig. 2. Top and bottom graphene are simulated with the same values of chemical potential and relaxation time. Using Eq. (5)^9,22^ and Eq.(6)^58,59^, it is possible to calculate the carrier density (*n*) and mobility of carriers ($\mu$) in each of the graphene layers simulated as

5 $$\begin{aligned} \begin{aligned} n=\frac{\mu _{c}^2}{\pi \hslash^{2}v_{f}^{2}}, \end{aligned}\end{aligned}$$

where $v_{f}$ is the Fermi velocity of $10^{6}$ m/s, and

6 $$\begin{aligned} \begin{aligned} \mu =\frac{e\tau v_{f}^{2}}{\mu _{c}}. \end{aligned}\end{aligned}$$

For the individual graphene layers used in the simulation of the graphene stack antenna and the single-layer graphene standard antenna, the sheet conductivity, the carrier mobility, and the carrier density achieve the values shown in Table 2. For comparison, a metallic antenna patch based on a 100-nm thick palladium (Pd) with a surface conductivity of 1.86 S is used for the simulation.

**Table 2:** Graphene sheet conductivity, carrier mobility, and carrier density of the graphene used for simulations with 1.2 ps of relaxation time and varying chemical potential.

Chemical potential (eV)	Sheet conductivity (S)	Mobility (cm$^2$/Vs)	Carrier density (cm$^{-2}$)
0.3	0.018	40,000	6.61$\times$ 10^12^
0.6	0.036	20,000	2.64$\times$ 10^13^
1.2	0.072	10,000	1.06$\times$ 10^14^

The chosen values of chemical potential have been selected since they can be achieved with a dielectric tuning within the configuration of the graphene stack antenna. The conductivity and carrier mobility of the graphene sheet presented in Table 2 are the ones related to Fig. 2a, b assuming a fixed chemical potential of 1.2 ps. In these figures, we provide a simplified analysis of how the chemical potential directly influences the resonant frequency of the graphene antenna. As a matter of fact, as the chemical potential increases, we expect a decrease in relaxation time^60,61^; however, this factor is not considered in the simulations discussed in this paper.

Indeed, the resonance frequency of graphene antennas can be predicted by the Eq. (7)^62^, which relates the plasmon wavevector to the graphene conductivity.

7 $$\begin{aligned} \begin{aligned} \frac{1}{\sqrt{\beta ^2-\frac{\omega ^2}{c^2}}}+\frac{\epsilon }{\sqrt{\beta ^2-\epsilon \frac{\omega ^2}{c^2}}}=\frac{-i\sigma }{\omega \epsilon _0}, \end{aligned}\end{aligned}$$

where $\beta$ is the plasmon wavevector, *c* is the light velocity, $\epsilon$ is the dielectric constant of substrate and $\epsilon _0$ is the vacuum permittivity constant. With a change in chemical potential, and consequently in conductivity, the plasmon wavevector is modified, and so the resonance frequency of graphene antennas. The real part of graphene’s conductivity directly influences the imaginary part of $\beta$, which defines the attenuation loss of the surface plasmon polariton wave. Conversely, the imaginary part of graphene’s conductivity directly affects the real part of $\beta$, which determines the wavelength of the SPP^63,64^.

From the simulation results, it is possible to observe the advantage of the proposed antenna over a standard monolayer antenna. The graphene stack configuration presents the same result as a standard monolayer graphene antenna but with **half** of the chemical potential (for the same relaxation time value). It means that the lower conductivity of the graphene due to the comparatively poor material quality of graphene produced by integratable manufacturing routes can be compensated by the stack configuration to a significant extent, e.g. from a sheet conductivity of 0.072 S for standard monolayer graphene with 1.2 eV to a sheet conductivity of 0.036 S for the stack configuration with graphene of 0.6 eV. Additionally, the graphene chemical potential, which is directly related to the graphene conductivity, will define the resonating frequency of the graphene antenna, i.e. the higher the chemical potential, the higher the resonance frequency.

As compared to the metallic antenna, which resonates at the designed frequency of 280 GHz, the resonance shift of the graphene antennas to lower frequencies is clearly visible, showcasing the possible reduction in size to achieve resonance at the same frequency value. For sub-micro-sized antennas, working at the higher frequency range, this frequency shift can reach even up to two orders of magnitute^62^, when graphene material quality conditions are met. The efficiency of the graphene antennas, however, is still lower compared to the metal antenna, but the stack configuration allows for an improvement on the realized gain.

### Antenna fabrication

The experimentally exhibited antennas are fabricated on a 50 $\mu$m Kapton polyimide layer on a silicon substrate, to demonstrate a fabrication process close to standard BEOL silicon microelectronics fabrication. A pre-treatment of the flexible Kapton polyimide film is necessary for the device fabrication. Annealing Kapton foil higher than 300 °C in vacuum conditions for at least one hour reduces deformation problems, which would otherwise cause misalignment issues during the later lithography processes. A layer of polydimethylsiloxane (PDMS) is introduced to adhere Kapton onto a standard silicon (Si) substrate throughout the fabrication process. The mixed PDMS solution is spin-coated on the Si substrate, and after curing, the annealed Kapton substrate is placed carefully on the platform. Additional metal antennas and graphene stack antenna samples on SiO_2_/Si as substrate are prepared as reference samples to the antenna measurements and to investigate all the processing steps, since 2D materials cannot be visualized on Kapton with an optical microscope. Besides, the Raman signal from graphene is usually hidden by the stronger background signal from the Kapton substrate.

**Fig. 3:**
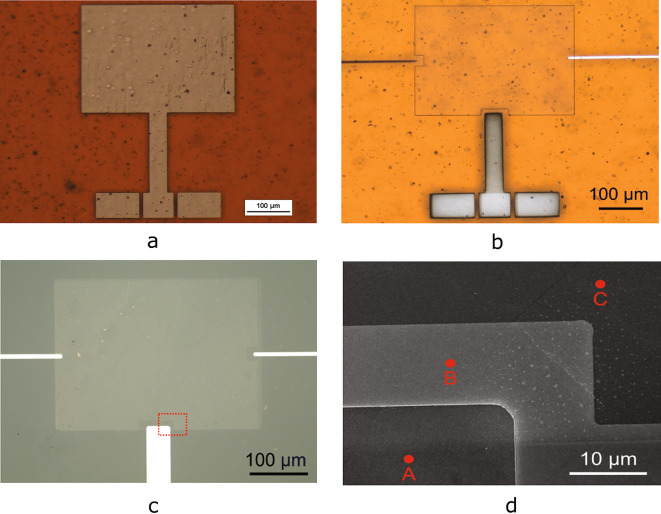
(**a**) Metal antenna reference on a polyimide substrate. (**b**) Graphene stack patch antenna on polyimide substrate. (**c**) Graphene stack patch antenna on the SiO_2_/Si—reference sample. (**d**) SEM investigation of the reference graphene stack antenna on the dashed line box in Fig.3c, where point A is the bottom graphene with contact with the signal line, point B is the bottom graphene covered with the dielectric layer, and point C is the second layer graphene on top the dielectric.

PMMA polymer-assisted wet transfer is used to transfer large-area CVD-grown 2D materials^21^. The graphene sheet is first transferred onto a hBN/Cu foil, and then the graphene/hBN stack is transferred to the Kapton substrate. This approach reduces the PMMA residues at the interface between graphene and hBN. Photolithography is used to open the resist window for the contact area to graphene, and then graphene is etched in the open area^65–67^. Afterward, 25 nm nickel (Ni) and 100 nm Pd are evaporated onto the sample and followed by a lift-off process to define the metal structures. An additional photolithography and Reaction-Ion Etching (RIE) oxygen plasma etching steps are used to pattern the bottom graphene patch. A metal line contacting the left edge of the bottom graphene sheet (see Fig. 3b) and the signal line are used as terminals for measuring the graphene resistance when it is placed directly on Kapton and when it is placed on the hBN layer. The measured resistances, which are $12\,{\textrm{k}}\Omega$ and $3\,{\textrm{k}}\Omega$, respectively, can be used as input parameters to obtain the graphene sheet resistance in the electromagnetic simulator COMSOL. The results indicate the sheet resistances are $1600\,\Omega /\square$ for graphene on hBN and $6400\,\Omega /\square$ for graphene on Kapton, and the corresponding sheet conductivities are on the order of ${6.5 \times 10^{-4}}$ S and ${1.56 \times 10^{-4}}$ S, respectively (under the assumption of negligible contact resistance). The decrease in resistance can be attributed to either the improvement of the interface graphene/hBN, or the change of doping and consequently Fermi level of graphene. It is worth mentioning, however, that the sheet resistance of graphene will present a strong variation depending on the device structure and encapsulation.

Subsequently, a layer of 80 nm Al_2_O_3_ is deposited on top of the bottom graphene patch. This thickness is chosen so that the dielectric is thin enough to enable the electromagnetic high-frequency coupling between the top and bottom graphene sheets and to enable the DC bias top-gate tuning of the bottom graphene sheet, but thick enough to avoid a leakage current between two graphene layers. Due to the chemically inert surface of graphene^68,69^, atomic layer deposition (ALD) of directly grown Al_2_O_3_ would present uniformity problems. Thus, a 2 nm evaporated aluminum (Al) is naturally oxidized in air to form a layer of Al_2_O_3_, which serves as a seed layer for the ALD deposition^68^. The electrical measurement between metal pads with high resistance can prove that Al is completely converted to Al_2_O_3_. Afterward, 80 nm ALD Al_2_O_3_ is deposited thermally on top of the bottom graphene.

Before transferring the second graphene layer, lithography, and buffered oxide etch (BOE) wet etching are necessary to open areas for probe contact on the GSG pads for the antenna measurements. After transferring the second layer of graphene, the same lithography and evaporation processes used for the first graphene to contact and RIE oxygen plasma etching to define the graphene patch are carried out. The final step is to peal the Kapton substrate from the PDMS/Si platform and deposit 200 nm Al on the back side of the Kapton substrate.

A metal line contacting the right edge of the top graphene and a bottom metal line contacting the left edge of the bottom graphene are used as terminals to measure the leakage current level between two graphene patches (Fig. 3b) and to bias the structure. The value measured is 10^-12^ A, which corresponds to the minimum possible measured value of the measurement setup, i.e. the top and bottom graphene layers are well isolated from each other.

To control all fabrication process steps, the graphene is additionally inspected on the SiO_2_/Si reference sample. As evident from Fig.3a, b, one can observe many defects on the polyimide surface. Due to the very thin layer of metal used and the graphene sheet, the entire antenna device will also present such surface defects replicating the polyimide substrate under it. This can affect the overall conductivity of the deposited metal and also the graphene conductivity used for the stack antenna.

### Antenna far-field measurement

**Fig. 4:**
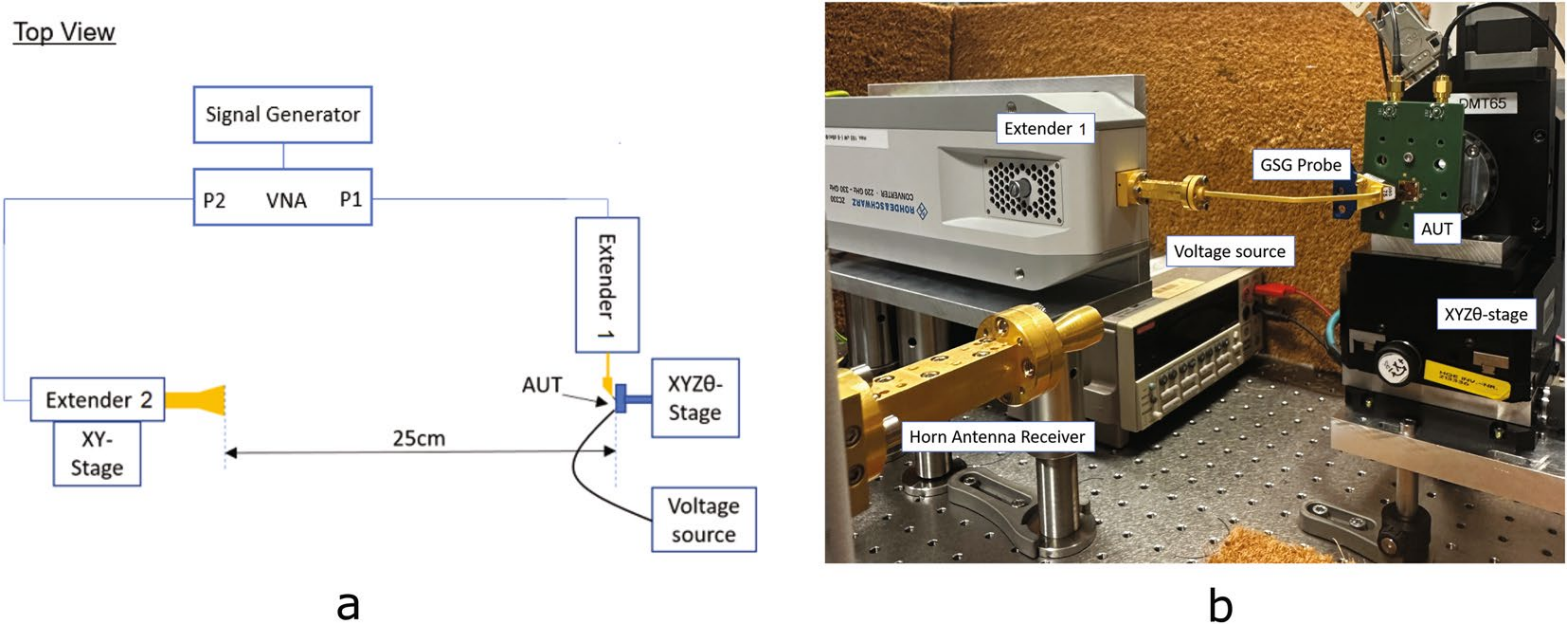
(**a**) Setup schematics showing the top view of the AUT far-field measurement setup used to characterize the graphene stack patch antennas and the metal patch antennas at the THz frequency range. (**b**) Picture of the measurement setup showing the mounted PCB board on an ${\textrm{XYZ}}\uptheta$ stage with the AUT contacted by the GSG probe connected to the frequency extender. A standard horn antenna is connected to the second frequency converter distant 25 cm from the AUT. Coconut doormat sheets, which have been characterized to have a reflection loss > 40 dB , are used as absorber material to ensure a proper anechoic measurement environment.

In order to characterize the proposed antenna, an antenna-under-test (AUT) far-field setup is constructed based on the schematics of Fig 4. A Rohde  & Schwarz vector network analyzer (VNA) of model ZVA67 with a Rohde  & Schwarz signal generator of model SMF100A are connected to two Rohde  & Schwarz frequency converters of model ZC330 at ports 1 and 2 to extend the originally operating frequency range from 10 MHz–67 GHz to 220–325 GHz and to obtain the S-parameters of the antenna. Port 2 is connected to a standard horn antenna (25 dBi gain) to act as the receiver antenna and port 1 is connected with a GSG Formfactor Infinity probe of model I325BT with 75$\upmu$m pitch (4 dBi loss) to contact the GSG pads of the AUT, which acts as the emitter antenna. The distance between the horn antenna and the AUT is 25 cm and the measurement is done in the bore sight direction of the antenna, i.e. at a normal incidence to the AUT. Graphene stack antennas, as well as metal antennas with the same dimensions, are measured and compared.

**Fig. 5:**
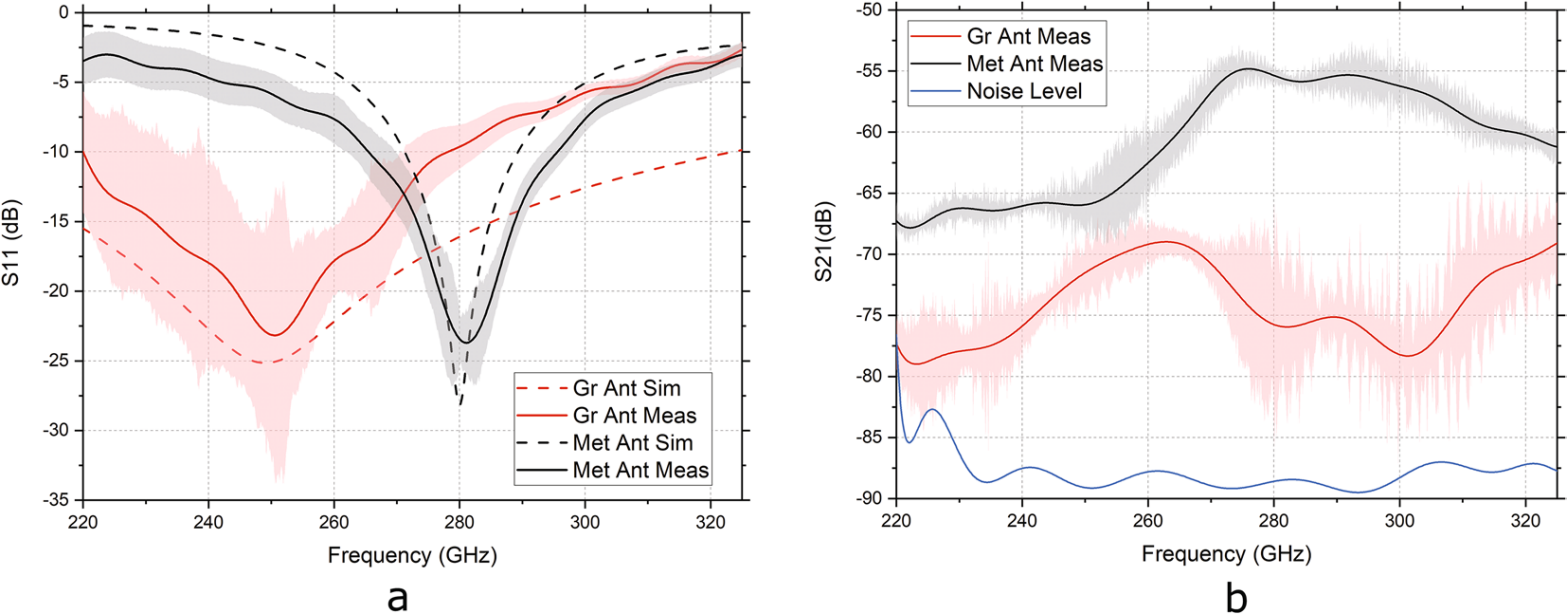
(**a**) S11 average measure (solid line) and simulated (dashed line) for the graphene antennas (red) and the metal antennas (black). The simulated graphene antenna had an impedance of $280+280i\,\Omega$. The shadow in both measured curves represents the calculated deviation of the S11 measured values for each case. (**b**) S21 average measure for the graphene antennas (red) and the metal antennas (black). The shadow in both measured curves represents the calculated deviation of the S21 measured values for each case. The blue line is the noise level of the measurement setup.

Before measurements, the open-short-load calibration procedure is performed on port 1. The average reflection coefficient measured for both graphene and metal antennas is shown in Fig. 5a. There is a good match between simulation and measurement results for the metal antenna with a small variation in the S-parameters measured between samples. The measured resonance frequency of 281.1 GHz and S11 of −23.7 dB agrees well with the simulated −28.14 dB at 280.1 GHz. This proves the robustness of the antenna design and the measurement setup. As for the graphene antennas, the average of S11 is similar to a simulated graphene stack with a graphene impedance of $280+280i\,\Omega$. Simulated S11 is −24.1 dB at 249.9 GHz versus measured values of −23.1 dB at 250.7 GHz. The antennas show a very good standing wave ratio of 1.15 and 1.14 for the measured graphene antenna stack and metallic antenna, respectively. The measured values, for the graphene antenna, are reasonably close to the simulated values but with slightly higher values of S11 and increased difference between simulation and measurement for the higher frequencies. There is a big S-parameters variation between the seven graphene antenna samples measured, which can be observed by the measurement error bar plotted. Due to the mechanical transfer processes of graphene and hBN, a graphene sheet can present a very different material quality between samples and even between different areas of the same sample. This translates to different graphene conductivities and, consequently, to the different antenna behaviors measured. Nevertheless, the graphene antenna presents the expected resonance behavior, which is markedly at a lower frequency compared to the metal antenna and the average measured value of resonance is close to the simulation value.

The S21 transmission coefficient measured for both antennas is present in Fig. 5b. The higher radiation of the graphene antenna at around 260 GHz can be seen clearly with a S21 of -69 dB versus the metal antenna at around 280 GHz with S21 of -55 dB. Even with a graphene stack, the proposed antenna presents a lower emission than a metal antenna probably due to high internal losses in the graphene patches. With a future enhancement of the fabrication processing, graphene with higher conductivity will compensate for the low antenna emission^45^. The current proposed antenna design with two graphene layers separated by a dielectric can compensate, to a meaningful extent, for the low graphene quality. A further improvement in CVD graphene manufacturing is still needed to reach the emission performance of classical low-loss metal antenna, which still is a challenge in the community.

**Fig. 6:**
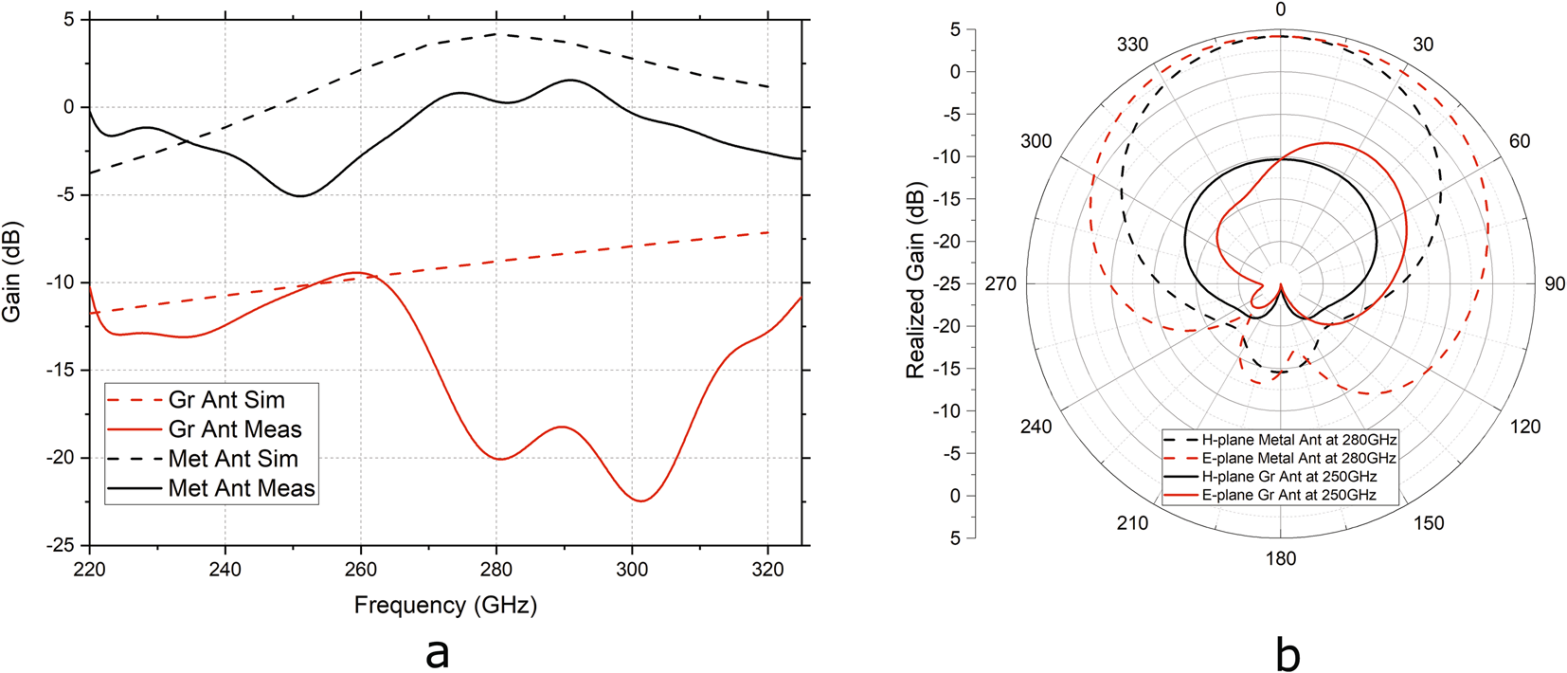
(**a**) Calculated (solid line) versus simulated (dashed line) gain of the graphene stack (red) and metal (black) antennas at a normal incidence to the AUT over the measurement frequency range of 220–325 GHz. (**b**) Simulated antenna H-plane (black) and E-plane (red) radiation patterns of the graphene stack (solid line) and metal (dashed line) antennas at their resonance frequency.

The gain of the antennas is shown in Fig. 6. The measured gain (Fig. 6a) is calculated based on the antenna gain transfer method using the values of S21 measured with the AUT as the emitter and with a previously done reference measurement with a standard horn antenna as the emitter. There is a mismatch between simulation and measurement gains for both metal and graphene antennas. Even though the S11 of the metal antenna matches well with the simulations, the calculated gain is lower than expected. For the simulated gain of the metal antenna, a peak at 280 GHz of 4.7 dB is present, while measurements show a gain of around 1.5 dB. This can be explained by additional internal losses in the metal due to the surface roughness of the polyimide substrate (see Fig. 3) and by reflections of the metal parts present in the measurement setup that can tilt the main lobe direction.

As for the graphene antenna, the inside losses are even higher and are also explained by the restricted graphene quality and probably tilted radiation angle. The simulated gain ranges from −11.7 dB to −7.2 dB within the measurement frequency range while, from the measurements, the gain matches simulation from 250  to 262 GHz and is lower at other frequencies for the graphene stack antenna. The simulated gain follows a nearly straight line and does not show increased gain values at the resonance point. In the simulation, the combination of real and imaginary impedances used to model graphene—chosen to fit the experimental data—behaves more like an effective absorber layer rather than exhibiting the expected radiation characteristics of a classical antenna. In contrast, the measurements indicate an increase in emission around the resonance frequency of the graphene stack antennas, demonstrating that the antenna is indeed emitting radiation at this frequency. Due to the limitations of the measurement setup, it is not possible to measure S21 and, consequently, gain of the antennas at different angles.

The H-plane and E-plane simulated radiation patterns of the metal and graphene antenna at their resonance frequencies can be seen in Fig. 6b. The metal antenna and the graphene antenna have a broad radiation angle. But the main lobe direction of the metal antenna in the E-plane is at 0°, while for the simulated graphene stack of $280+280i\,\Omega$ the lobe direction is at 36°. The main lobe direction of the graphene antenna in the E-plane will be dependent on the graphene conductivity. Low conductivities of graphene lead to a lower radiation gain that can be easily tilted by the high-conductive metal microstripline.

## Discussion

A CVD graphene stack patch antenna was proposed, designed, simulated, manufactured, characterized and proved to emit THz radiation in the frequency range of 220–325 GHz. Our proposed antenna resonates at 250.7 GHz with a gain value of −9.5 dB measured at $0^{\circ }$ incidence angle. Based on simulations, this value can even reach −6.7 dB when the main lobe direction of emitted radiation (36°) is considered. It should be emphasized that this restricted emission efficiency is bound to the first experimental realization of such a graphene antenna. The emission efficiency will improve significantly with the graphene conductivity and will also directly influence the resonance frequency of the antenna (see Fig. 2). The big variation in the graphene conductivity makes it difficult to predict the precise resonance behavior and matching of real fabricated antennas. Furthermore, the presence of two separately processed graphene patches with a large graphene area encompassing inhomogeneous areas of different conductivities makes the simulation predictions and the definition of the real values of chemical potential and relaxation time of the graphene used extremely difficult. Due to these reasons, the impedance values used to simulate the graphene stack antennas measured can only be considered as a rough approximation of the real values of the graphene material used. Although the emission efficiency of the graphene antennas presented here is low, it is the first real experimental demonstration of emission from graphene antennas at THz range using monolayer CVD graphene. Widely available simulation results of many publications assume values for the graphene characteristics that are practically unrealistic (e.g. 1 ps of relaxation time^28,29,31^), which is not the case for the real measurements using CVD monolayer graphene demonstrated here. Despite this fact, we could find a value of graphene impedance that translates into a similar behavior to the one observed during measurements for the graphene antenna case, at least for the low frequencies of the measurement range.

The large standard variation in the measurement data is due to the already mentioned significant variation between different graphene samples. Although we could better match the simulation and measurements for S11, this did not translate into a good match between simulated gain and measured gain. Some possible explanations can be the increased internal losses in the metal patch, as well as in the graphene, which was not considered for the simulations. Further investigation of the real graphene conductivity on this configuration is necessary to fully understand the emission characteristics of the graphene stack antenna and define the ideal impedance values that will perfectly match measurements and simulations. We also observed a small mismatch for the measured gain of the metal antenna. This is mainly caused by the roughness of the polyimide substrate, which can lead to increased internal losses in the metal, and also by reflections of the measurement setup.

A full radiation pattern characterization is needed to point out what is, in fact, the main lobe direction of the AUT. At $0^{\circ }$ normal incidence to the antenna, the metal antenna provides a high emission in the region of its resonance frequency, as well as the graphene antenna. The graphene antenna emission is still lower than a metal antenna emission and high-quality graphene is needed for improved antenna emission results.

One should emphasize, that despite limited performance, this graphene stack antenna does provide a significant improvement in the radiation characteristics of graphene antennas. Single-layer graphene patches fabricated with an identical processing procedure did not yield any measurable emission. Only the stacked bilayer patch structure proposed here did improve emission characteristics sufficiently, to warrant a measurable emission characteristic, despite the finite performance of CVD graphene material processed and integrated on an organic and rough substrate such as polyimide. Additionally, the size reduction possibility when using graphene antennas is proven experimentally. In our case, the possible size reduction is in the range of 10%, but it can be orders of magnitude smaller than a standard metallic antenna at the higher frequencies of the spectrum^62^.

## Conclusion

In this work, we demonstrated the first graphene stack patch antenna fabricated with monolayer CVD graphene working at the THz frequency range of 220–325 GHz. Due to the special characteristics of graphene surface plasmons, the graphene antenna demonstrates a significantly lower resonance frequency, 10 % reduction, than the metal antenna of the same dimensions. This size reduction (relative to the resonance wavelength) experimentally proves the higher integration potential of graphene antennas in comparison to classical metallic antennas. This advantage of the proposed antenna allows the future reduction of the transceiver chip sizes for future communication components. An increased THz emission by the graphene antenna is achieved by the stack configuration in comparison to a monolayer patch antenna, as predicted by simulations. The planar antenna and the excitation geometry presented here permit easy integration of the proposed antenna with current CMOS technologies as a BEOL process. An improved result for the efficiency and the far-field gain will become possible with higher quality graphene samples, as it is directly related to the graphene conductivity. As a plethora of research groups and the semiconductor industry are investing in scalable higher-quality and reproducibility graphene manufacturing processes, we are confident that graphene antennas will reach the performance of metallic antennas in the foreseeable future, while retaining its unique advantages of compatibility, miniaturization and reconfigurability.

## Methods

### Device fabrication

A 50 µm thick polyimide Kapton film with dielectric constant 3.4 at 10 GHz and tangent loss 0.002 from Manufacturer DuPont was used as substrate for the antennas. A mix of 10:1 of PDMS and curing agent from Sylgard 184 was used as an adhesion layer between the polyimide and a standard substrate holder. CVD graphene and hBN were from General Graphene.

### COMSOL simulation

The simulation software COMSOL Multiphysics version 6.2 was used to simulate the electrical field and potential distribution within the graphene and metal electrodes. The sheet resistance and sheet conductivity of the graphene patch can be determined by the simulation geometry factor result and the resistance of the graphene measured between two metal contact electrodes.

### HFSS simulation

The 3D electromagnetic simulation software ANSYS HFSS version 2023.1.0 was used to simulate the antennas. The modal analysis was used to simulate both metal and graphene antennas. Both antennas were of the same dimensions and a waveguide port was used to excite the antennas. To match measurements, polyimide used for simulations had a dielectric constant of 3.1 and tangent loss of 0.0055. The graphene was simulated as a 2D sheet with impedance boundary condition calculated from the Kubo formula over the simulation frequency range. Driven solution setup analysis at multiple frequencies was performed with a maximum delta set to 0.001.

### VNA measurements

For the gain calculations, a reference measurement was performed with standard horn antennas as emitter and receiver. The distance between the antennas plane was 25 cm. Before measurements of the AUT, a short-open-load (SOL) calibration routine was performed using the calibration substrate model CS-15 from GGB Industries. An ${\textrm{XYZ}}\uptheta$ stage was used to place the AUT in contact with the GSG probe. Two cameras were used for better visualization of the contact between the GSG probe and GSG pads (one looking at the GSG probe from the top and another with the GSG probe front view). A total of four metal antennas and seven graphene stack antennas were measured. Each measurement was done 100 times with 1001 measuring points within the 220 - 325 GHz frequency range. Self-written LabView and MATLAB scripts were used to acquire the S-parameters data and process the measured curve.

## Supplementary Information

Supplementary Information.

## Data Availability

The datasets generated during and/or analyzed during the current study are available from the corresponding author upon reasonable request.
