# Supplementary material for: A THz graphene-on-hBN stack patch antenna for future 6G communications
Source: Sci Rep. 2025 Sep 23;15:32650. doi: 10.1038/s41598-025-16695-x (PMC12457587; doi:10.1038/s41598-025-16695-x)
Supplement: Supplementary file 1 — Supplementary Information. [file 41598_2025_16695_MOESM1_ESM.pdf]

A THz Graphene-on-hBN Stack Patch Antenna for Future 6G Communications (Supplementary Information)

Elana P. de Santana[1,*], Kun-Ta Wang[2,3], Sergi Abadal[4], Daniel Stock[1], Zhenxing Wang[2], Anna-Katharina Wigger[1], Eduard Alarcón[4], Max C. Lemme[2,3], and Peter Haring Bolívar[1]

[1]University of Siegen, Institute of High Frequency and Quantum Electronics, Siegen, 57076, Germany
[2]AMO GmbH, Advanced Microelectronic Center Aachen (AMICA), Aachen, 52074, Germany
[3]RWTH Aachen University, Chair of Electronic Devices, Aachen, 52074, Germany
[4]Universitat Politècnica de Catalunya, NanoNetworking Center in Catalunya (N3Cat), Barcelona, 08034, Spain
[*]elana.psantana@uni-siegen.de

1. Patch Antenna Dimensions Calculation

In this section, we show the antenna dimensions calculations in detail.

The proposed antenna dimensions are defined for a resonance at f_r = 280 GHz.

Initial parameters are chosen before calculations, such as the dielectric constant (ε_r) of the substrate to be used (polyimide substrate with ε_r = 3.5) and its thickness (t = 50 µm).

All antenna dimensions calculations are shown in Equations 1-5 below, where W_p is the antenna patch width, ε_{eff} is the effective dielectric constant, L_{eff} is the effective antenna patch length, ΔL_p is the antenna patch length extension, and L_p is the antenna patch length.

$$W_p = \frac{c}{2f_r\sqrt{\frac{\varepsilon_r + 1}{2}}} \approx 357\mu m \tag{1}$$

$$\varepsilon_{eff} = \frac{\varepsilon_r + 1}{2} + \frac{\varepsilon_r - 1}{2}\left[1 + 12\frac{t}{W_p}\right]^{-1} \approx 3 \tag{2}$$

$$L_{eff} = \frac{c}{2f_r\sqrt{\varepsilon_{eff}}} \approx 309 \tag{3}$$

$$\Delta L_p = 0.412 t_s \frac{\left(\varepsilon_{eff} + 0.3\right)\left(\frac{W_p}{t} + 0.264\right)}{\left(\varepsilon_{eff} - 0.258\right)\left(\frac{W_p}{t} - 0.8\right)} \approx 23\mu m \tag{4}$$

$$L_p = L_{eff} - 2\Delta L_p \approx 262\mu m \tag{5}$$

The calculated dimensions lead to an antenna with a resonance frequency at 280 GHz. By substituting the metal patch with a graphene patch, the resonance frequency is shifted to lower values.

2. Graphene Conductivity Model - Theory

In this section, we derive the equation for graphene conductivity based on Kubo's linear response theory. In this classical derivation[1,2,3], the frequency-dependent electrical conductivity tensor $\sigma_{\alpha\beta}(\Omega)$ is defined as

$$\sigma_{\alpha\beta}(\Omega) = \frac{K_{\alpha\beta}(\Omega+i0)}{-i(\Omega+i0)} \,, \qquad (6)$$

with

$$K_{\alpha\beta}(\Omega + i0) \equiv \frac{<\tau_{\alpha\beta}>}{V} + \frac{\Pi^{R}_{\alpha\beta}(\Omega+i0)}{\hbar V} \,, \qquad (7)$$

Where $\sigma_{\alpha\beta}(\Omega)$ is the graphene conductivity in an arbitrary $\alpha\beta$ direction, Ω is the frequency of the electrical excitation, V is the volume of the system, $\Pi^{R}_{\alpha\beta}$ is the retarded current-current correlation function, and $< \tau_{\alpha\beta} >$ is the stress tensor.

Further solving the above equations, considering an applied magnetic field $B \rightarrow 0$ [2], the graphene conductivity reduces to only the longitudinal conductivity $\sigma_{xx}(\Omega)$

$$\sigma_{xx}(\Omega) = -\frac{2ie^2(\Omega+2i\Gamma)}{h}\left[\frac{1}{(\Omega+2i\Gamma)^2}\int_{\Delta}^{\infty} d\omega\ \frac{\omega^2-\Delta^2}{\omega}\left(\frac{\partial n_F(\omega)}{\partial\omega} - \frac{\partial n_F(-\omega)}{\partial\omega}\right) - \int_{\Delta}^{\infty} d\omega\ \frac{\omega^2+\Delta^2}{\omega^2}\ \frac{n_F(-\omega)-n_F(\omega)}{(\Omega+2i\Gamma)^2-4\omega^2}\right] \,, \qquad (8)$$

with h being Planck's constant, Γ being the scattering rate, n_F being the Fermi-Dirac distribution for electrons, ω is the frequency proportional to energy, and Δ being an excitonic gap included in the Landau level energies.

Considering an approximation with $\Delta = 0$, the graphene conductivity reads

$$\sigma_{xx}(\Omega) = -\frac{2ie^2(\Omega+2i\Gamma)}{h}\left[\frac{1}{(\Omega+2i\Gamma)^2}\int_{0}^{\infty} d\omega\ \omega\left(\frac{\partial n_F(\omega)}{\partial\omega} - \frac{\partial n_F(-\omega)}{\partial\omega}\right) - \int_{0}^{\infty} d\omega\ \frac{n_F(-\omega)-n_F(\omega)}{(\Omega+2i\Gamma)^2-4\omega^2}\right] \qquad (9)$$

Now, we rename the variables from the original formulation[1,2,3] to the more commonly used variables as indicated in Supplementary Table 1 below.

Original variable	Updated variable
$\sigma_{xx}(\Omega)$	$\sigma(\omega)$
Ω	ω
2Γ	$1/\tau$
$n_F(\omega)$	$f_d(E)$
ω	$E/\hbar$
$d\omega$	$dE/\hbar$

Supplementary Table 1: Renaming variables of the graphene conductivity equation

The rewriting of Eq. 9 is done in three parts. First, we rewrite the pre-factor $-\frac{2ie^2(\Omega+2i\Gamma)}{h}$, then the first term inside the square brackets, and finally the second term inside the square brackets.

The pre-factor transforms into

$$-\frac{2ie^2(\Omega+2i\Gamma)}{h} = -\frac{2ie^2\left(\omega+\frac{i}{\tau}\right)}{2\pi\hbar} = -\frac{ie^2\left(\omega+\frac{i}{\tau}\right)}{\pi\hbar}\ , \tag{10}$$

with h=$2\pi\hbar$.

The first term inside the square brackets transforms into

$$\frac{1}{(\Omega+2i\Gamma)^2}\int_0^\infty d\omega\ \omega\ \left(\frac{\partial n_F(\omega)}{\partial\omega} - \frac{\partial n_F(-\omega)}{\partial\omega}\right) = \frac{1}{\left(\omega+\frac{i}{\tau}\right)^2}\int_0^\infty \frac{dE}{\hbar}\ \frac{E}{\hbar}\ \left(\frac{\partial f_d(E)\hbar}{\partial E} - \frac{\partial f_d(-E)\hbar}{\partial E}\right)\ , \tag{11}$$

which reduces to

$$\frac{1}{\left(\omega+\frac{i}{\tau}\right)^2}\frac{1}{\hbar}\int_0^\infty dE\ E\ \left(\frac{\partial f_d(E)}{\partial E} - \frac{\partial f_d(-E)}{\partial E}\right)\ . \tag{12}$$

The second term inside the square brackets is expressed as

$$\int_0^\infty d\omega\ \frac{n_F(-\omega)-n_F(\omega)}{(\Omega+2i\Gamma)^2-4\omega^2} = \int_0^\infty \frac{dE}{\hbar}\ \frac{f_d(-E)-f_d(E)}{\left(\omega+\frac{i}{\tau}\right)^2-4\left(\frac{E}{\hbar}\right)^2}\ . \tag{13}$$

Combining Eq. (10), (12) and (13), we have the graphene conductivity equation presented in the main manuscript as Eq. 1, which is

$$\sigma(\omega) = -\frac{ie^2\left(\omega+\frac{i}{\tau}\right)}{\pi\hbar^2}\left[\frac{1}{\left(\omega+\frac{i}{\tau}\right)^2}\int_0^\infty \left(\frac{\partial f_d(E)}{\partial E} - \frac{\partial f_d(-E)}{\partial E}\right) E dE\ - \int_0^\infty \frac{f_d(-E)-f_d(E)}{\left(\omega+\frac{i}{\tau}\right)^2-4\left(\frac{E}{\hbar}\right)^2} dE\right]\ , \tag{14}$$

with Fermi distribution described as

$$f_d(E) = \frac{1}{e^{\frac{E-\mu_c}{k_B T}}+1}\ , \tag{15}$$

with k_B being the Boltzmann constant, μ_c being the chemical potential and T being the temperature.

In Eq. (14), both intraband and interband transition contributions to the graphene conductivity are presented [1,2,3]. The first term of Eq. (14) is considered as the intraband term due to the presence of the derivative of the Fermi-Dirac distribution, which accounts for the available states near the Fermi level, with low energy. On the other hand, the second term is considered as the interband contribution due to the presence of $f_d(-E) - f_d(E)$, which corresponds to the difference between the states of the valence and conduction band, which requires higher energies.

We can simplify Eq. (14) to obtain only the intraband and interband conductivity equations.

Starting with the intraband term:

$$\sigma_{intra}(\omega) = -\frac{ie^2\left(\omega+\frac{i}{\tau}\right)}{\pi\hbar^2}\left[\frac{1}{\left(\omega+\frac{i}{\tau}\right)^2}\int_0^\infty dE\; E\,\left(\frac{\partial f_d(E)}{\partial E} - \frac{\partial f_d(-E)}{\partial E}\right)\right] . \quad (16)$$

After simplification,

$$\sigma_{intra}(\omega) = -\frac{ie^2}{\pi\hbar^2}\frac{1}{\left(\omega+\frac{i}{\tau}\right)}\left[\int_0^\infty dE\; E\,\left(\frac{\partial f_d(E)}{\partial E} - \frac{\partial f_d(-E)}{\partial E}\right)\right] . \quad (17)$$

The integral in Eq. 17 reduces to $-\mu_c + 2k_BT\ \ln\left(1 + \exp\left(\mu_c/(k_BT)\right)\right)$.

Then, knowing that

$$\ln\left(1 + e^{\frac{\mu_c}{k_BT}}\right) = ln\left(2cosh\left(\frac{\mu_c}{2k_BT}\right)\right) + \frac{\mu_c}{2k_BT} , \quad (18)$$

The intraband term for the graphene conductivity becomes

$$\sigma_{intra}(\omega) = \frac{2k_BTe^2}{\pi\hbar^2}\ ln\left(2cosh\left(\frac{\mu_c}{2k_BT}\right)\right)\frac{i}{\left(\omega+\frac{i}{\tau}\right)} . \quad (19)$$

Now for the interband term:

$$\sigma_{inter}(\omega) = -\frac{ie^2\left(\omega+\frac{i}{\tau}\right)}{\pi\hbar^2}\left[-\int_0^\infty dE\ \frac{f_d(-E)-f_d(E)}{\left(\omega+\frac{i}{\tau}\right)^2-4\left(\frac{E}{\hbar}\right)^2}\right] . \quad (20)$$

To solve this equation, we assume $T \rightarrow 0$, which will lead to

$$\sigma_{inter}(\omega) = \frac{ie^2\left(\omega+\frac{i}{\tau}\right)}{\pi\hbar^2}\left[\int_0^{\mu_c} dE\ \frac{1}{\left(\omega+\frac{i}{\tau}\right)^2-4\left(\frac{E}{\hbar}\right)^2}\right] . \quad (21)$$

The integral can be solved to

$$\int_0^{\mu_c} \frac{1}{\left(\omega+\frac{i}{\tau}\right)^2-4\left(\frac{E}{\hbar}\right)^2} dE = \frac{\hbar}{4\left(\omega+\frac{i}{\tau}\right)} ln\left(\frac{2\frac{\mu_c}{\hbar}+\left(\omega+\frac{i}{\tau}\right)}{2\frac{\mu_c}{\hbar}-\left(\omega+\frac{i}{\tau}\right)}\right) \quad . \tag{22}$$

Therefore, inserting Eq. 22 into Eq. 21, the simplified interband conductivity of graphene, assuming that $\hbar\omega \geq \mu_c$, can be described as

$$\sigma_{inter}(\omega) = \frac{ie^2}{4\pi\hbar} ln\left(\frac{2\mu_c+\left(\omega+\frac{i}{\tau}\right)\hbar}{2\mu_c-\left(\omega+\frac{i}{\tau}\right)\hbar}\right) \quad . \tag{23}$$

3. Graphene Conductivity Model - Simulation

In this section, we show the values of the graphene sheet conductivity over the THz spectral range, as well as in the range of the measurement setup from 220 GHz – 325 GHz.

The conductivity of the graphene sheet is determined using Eq. 3 from the main manuscript text. We only consider intraband contributions since interband contributions are negligible within this frequency range. Then, the graphene conductivity is calculated for 1000 frequency points within the THz range, which spans from 0.1 to 10 THz, as well as for 1000 frequency points in the range of interest of this paper, specifically between 220 and 325 GHz.

Initially, a fixed relaxation time of 0.5 ps is utilized, while the chemical potential is varied among 10 different values, ranging from 0.1 to 1 eV. Supplementary Figs. 1 and 2 illustrate the effect of the chemical potential on the conductivity values of graphene across the mentioned frequency ranges.

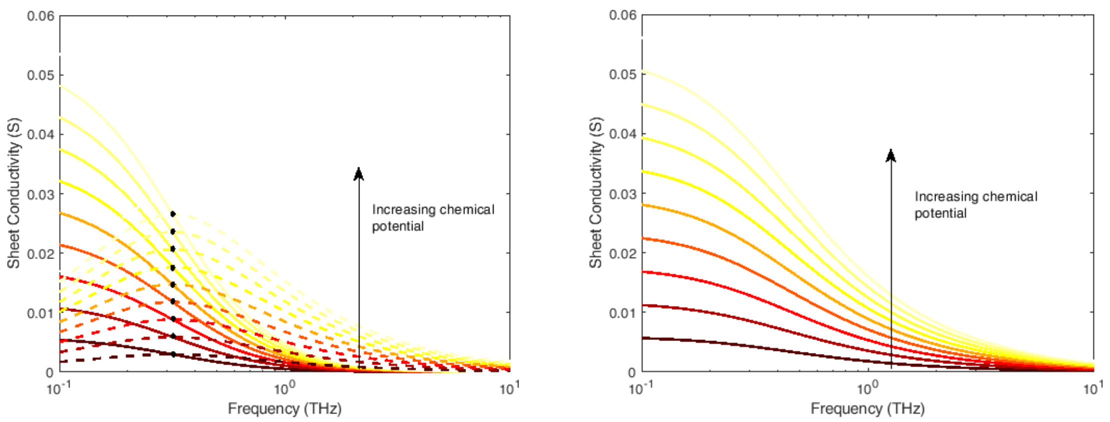

Supplementary Fig. 1: Graphene conductivity at the THz frequency range 0.1 – 10 THz with graphene of 0.5 ps of relaxation time and chemical potential from 0.1 eV to 1 eV. Left: real (solid line) and imaginary (dashed line) parts of the conductivity. Right: absolute value of conductivity. The black points in the left plot illustrate the frequency at which the imaginary part becomes higher than the real part.

There is an exponential decrease in the conductivity values of graphene across all chemical potentials as the frequency increases. Furthermore, both the real and imaginary parts of graphene's conductivity increase with higher chemical potential.

At lower frequencies within the THz range, the real part of the conductivity is greater than the imaginary part. As the frequency rises, the real part of the conductivity decreases while the imaginary part increases until they become equal. Beyond this point, the real part becomes smaller than the imaginary part. Although the imaginary part begins to decrease at this stage, it does so at a slower rate than the real part. The frequency point at which the real and imaginary parts of the graphene conductivity become equal, indicated by the black dot, is independent of the chemical potential values.

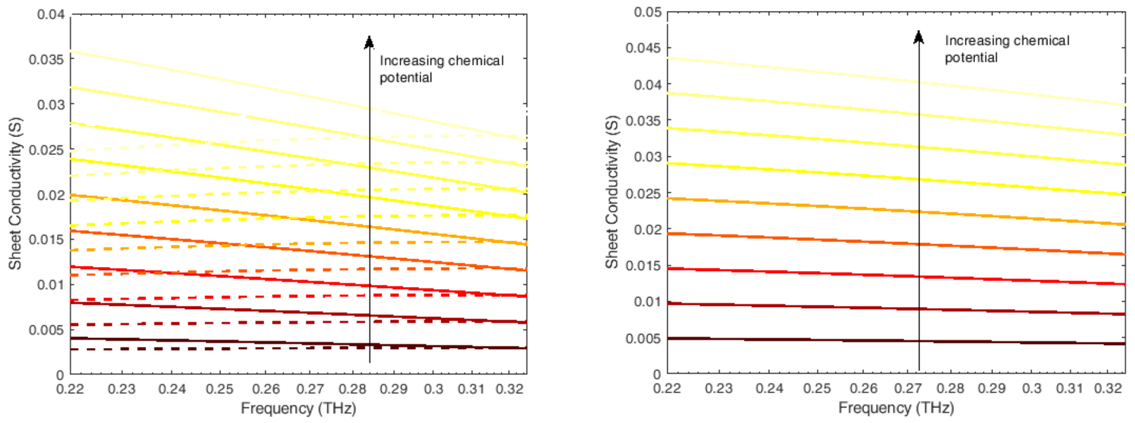

Supplementary Fig. 2: Graphene conductivity at the measurement frequency range 220 - 325GHz with graphene of 0.5 ps of relaxation time and chemical potential from 0.1 eV to 1 eV. Left: real (solid line) and imaginary (dashed line) parts of the conductivity. Right: absolute value of conductivity.

In the frequency range discussed in this paper, a relaxation time of 0.5 ps combined with 10 different chemical potential values ranging from 0.1 to 1 eV results in the real part of graphene conductivity consistently exceeding the imaginary part. As the chemical potential increases, the difference between the imaginary and real parts decreases.

Next, a fixed chemical potential of 1 eV is used, while the relaxation time is varied for 10 values within the range of 0.1 to 1 ps. Supplementary Figs. 3 and 4 illustrate the effect of the relaxation time on the conductivity values of graphene across the mentioned frequency ranges.

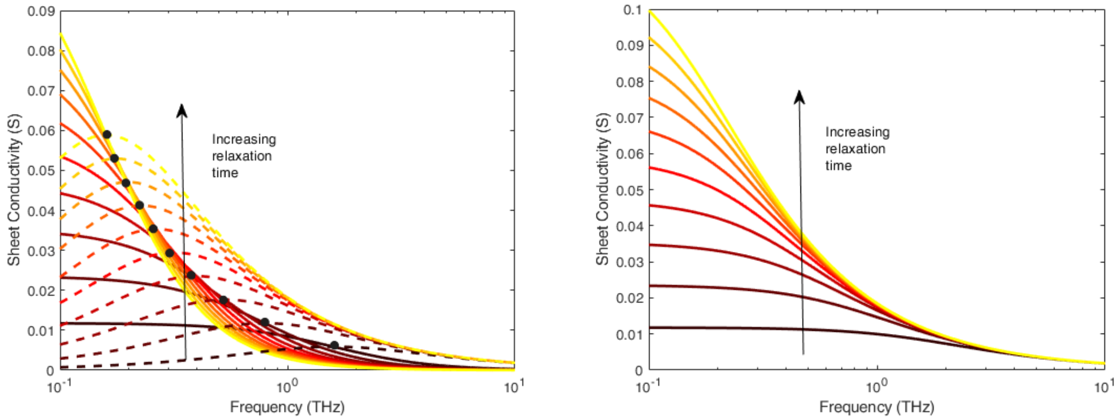

Supplementary Fig. 3: Graphene conductivity at the THz frequency range 0.1 – 10 THz with graphene of 1 eV of chemical potential and relaxation time from 0.1 ps to 1 ps. Left: real (solid line) and imaginary (dashed line) parts of the conductivity. Right: absolute value of conductivity. The black points in the left plot illustrate the frequency at which the imaginary part becomes higher than the real part.

There is an exponential decrease in the conductivity values of graphene across all relaxation times as the frequency increases. Furthermore, both the real and imaginary parts of graphene's conductivity increase with higher chemical potential.

As before, at lower frequencies within the THz range, the real part of the conductivity is greater than the imaginary part. As the frequency increases, the real part of the conductivity decreases while the imaginary part increases until they become equal. Beyond this point, the real part becomes smaller than the imaginary part. Although the imaginary part begins to decrease at this stage, it does so at a slower rate than the real part. It is important to note here that by increasing the relaxation time, the frequency at which the real and imaginary parts of the graphene conductivity become equal, indicated by the black dot, shifts to lower frequencies. With smaller values of chemical potential, the real part of the graphene conductivity stays constant through a larger range of frequencies before it starts to decrease.

For the current chemical potential and relaxation time combinations, for frequencies higher than 2 THz, the absolute value of graphene conductivity is not affected by the relaxation time.

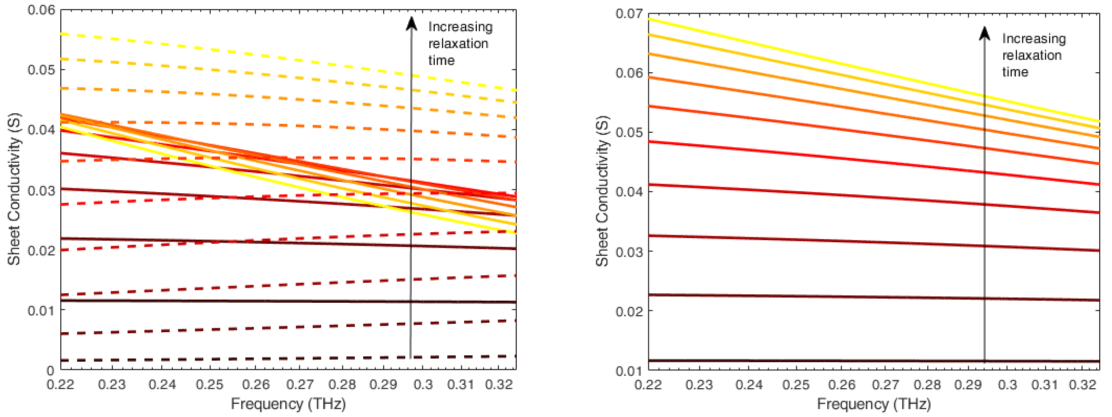

Supplementary Fig. 4: Graphene conductivity at the measurement frequency range 220 – 325 GHz with graphene of 1 eV of chemical potential and relaxation time from 0.1 ps to 1 ps. Left: real (solid line) and imaginary (dashed line) parts of the conductivity. Right: absolute value of conductivity.

In the frequency range examined in this paper, a chemical potential of 1 eV, combined with 10 different relaxation time values ranging from 0.1 to 1 ps, results in an increase in graphene's conductivity. The absolute conductivity begins to approach saturation, where further increases in relaxation time have little to no effect on the overall conductivity.

For low relaxation times at this frequency range, the imaginary part is smaller than the real part up to a relaxation time of 0.7 ps. Beyond 0.7 ps, the imaginary part of graphene becomes larger than the real part. At this point, the real part no longer increases with increasing relaxation time; instead, it exhibits a linear decrease over frequency, contrasting with its almost constant behavior observed for relaxation times smaller than 0.7 ps.

The graphene impedance values used to simulate the graphene stack patch antenna are calculated based on the conductivity values, such as:

$$Z_{re} = \frac{\sigma_{re}}{{\sigma_{re}}^2 + {\sigma_{im}}^2} \quad (24)$$

$$Z_{im} = \frac{-\sigma_{im}}{{\sigma_{re}}^2 + {\sigma_{im}}^2} \quad (25)$$

where, σ_{re} and σ_{im} are the real and imaginary parts of the graphene conductivity calculated with Eq. 3 of the main manuscript text.

The graph depicting the conductivity and impedance of graphene used for the simulation over the frequency range of 220 - 325 GHz is shown in Supplementary Fig. 5. The graphene utilized for the antenna simulation had a relaxation time of 1.2 ps and chemical potentials of 0.3 eV, 0.6 eV, and 1.2 eV.

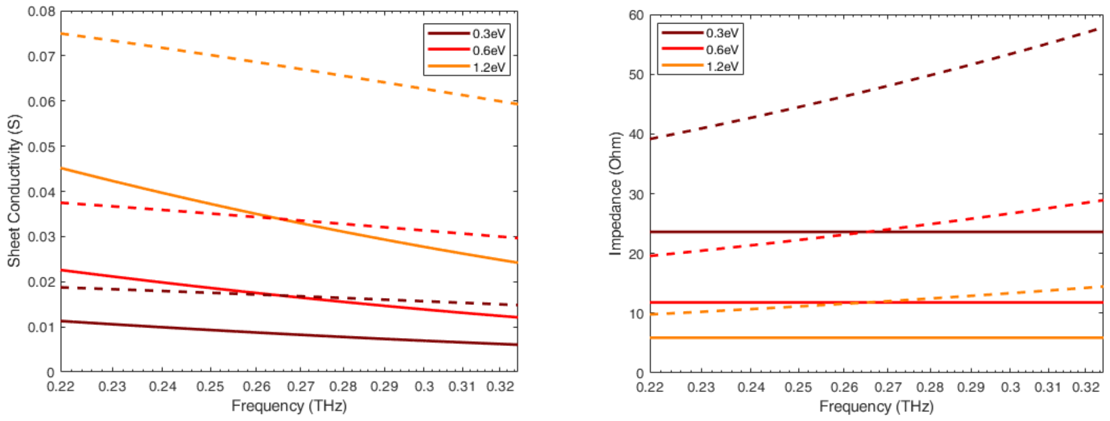

Supplementary Fig. 5: The real part (solid line) and the imaginary part (dashed line) of (a) the conductivity values of the graphene used in the antenna simulation, and (b) the impedance values of the graphene used in the antenna simulation.

4. <u>Measured S-parameters</u>

In this section, we show the raw measurement data (not processed) of the S-parameters of the metal and graphene antennas measured.

Supplementary Fig. 6 shows the average measurement of S11 and S21 of the metal and graphene antennas.

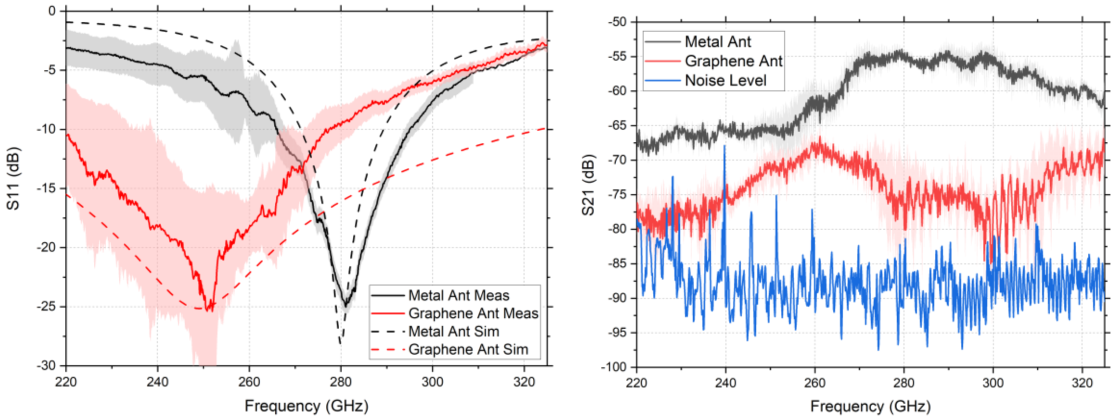

Supplementary Fig. 6: Average measurement of S-parameters of the metal antennas (black solid line) and graphene stack antennas (red solid line) and respective simulations (dashed lines). Left: Average of S11 measurement with the error bar (shadow area). Right: Average S21 measurement with the error bar (shadow area) and the noise level of the measurement setup (blue line).

The comparison between the measured S11 and S21 of the graphene stack antenna versus the metal antenna shows the superiority of the metal antenna over the graphene stack antenna. Not only does the metal antenna present the expected behavior of resonance at 280 GHz, but also its S21 value is clearly higher than the graphene stack antenna. Nevertheless, the measurement results of the graphene stack antenna show that the graphene antenna presents a resonance at 250 GHz, which indicates the possible reduction in antenna size, even though its emission is very low compared to the metal antenna.

Supplementary Fig. 7 shows measurements of S11 and S21 of all the metal antennas measured. It is possible to observe the behavioral similarity of the four antennas.

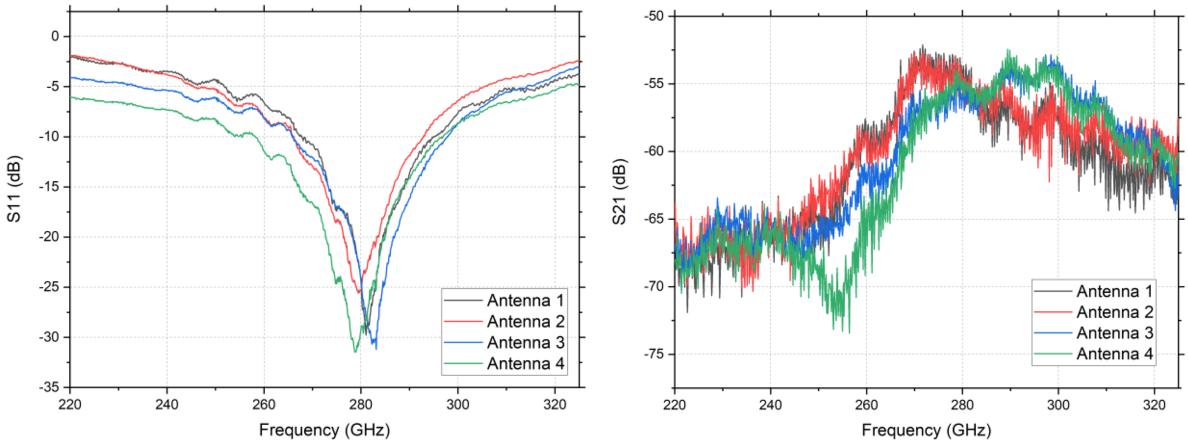

Supplementary Fig. 7: Measured S-parameters of the metal antennas. Left: S11 measurement. Right: S21 measurement.

The four metal antennas measured showed similar S11 values. In the S21 measurements, antennas 1 and 2 exhibited very similar performance. Antenna 3 behaved similarly to antennas 1 and 2 at lower frequencies but displayed a different pattern after 280 GHz, with higher emissions than antennas 1 and 2. In contrast, antenna 4 had lower emissions compared to the

other antennas up to 280 GHz; however, after this point, it produced results like those of antenna 3. The significant difference in S21 compared to S11 among the samples can be attributed to the different loss mechanisms present in each metal antenna sample. Although they receive power similarly, their power emission varies based on their internal losses.

On the other hand, Supplementary Fig. 8 shows the measurement of S11 and S21 of all the graphene antennas measured. It is possible to observe the different behaviors of the seven graphene stack antennas.

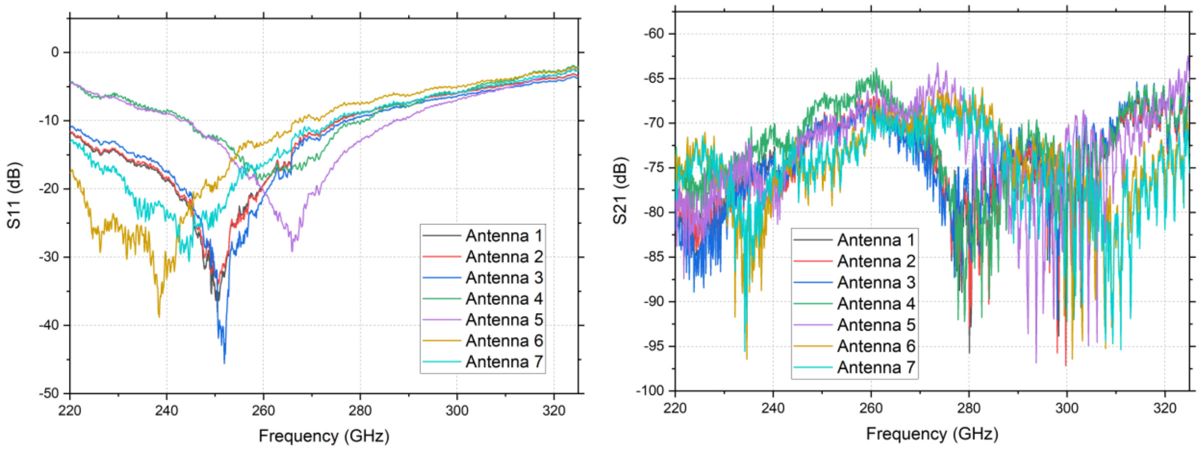

Supplementary Fig. 8: Measured S-parameters of the graphene antennas. Left: S11 measurement. Right: S21 measurement.

The seven graphene stack antennas measured exhibited different S11 values. Antenna 6 resonated at approximately 240 GHz, while antenna 7 resonated around 245 GHz. Antennas 1, 2, and 3 all resonated near 250 GHz, and antennas 4 and 5 showed a resonance at 268 GHz. There is significant variation among the graphene stack antennas, as the graphene material used for each sample differs, resulting in distinct antenna behaviors. Nevertheless, all graphene stack antenna samples demonstrated resonance frequencies that are lower compared to those of metal antennas.

In the S21 measurements, antennas 1, 2, 3, and 4 exhibited similar emission characteristics, with antenna 4 demonstrating a slightly higher emission. Meanwhile, samples 5, 6, and 7 also showed comparable behavior. In all cases, the higher S21 values occurred at the frequency point where there was a greater acceptance of input power, as indicated by the S11 measurement results. Additionally, the significant variation in the graphene material used for each antenna affects not only the S11 values and resonance frequency, but also directly impacts power emission. This is because the internal losses of each graphene antenna can differ considerably.

5. Graphene Stack Antenna Simulation Gain Plots

In this section, we present the efficiency plots for the simulated antennas shown in Figs. 2(a) and 2(b) of the main manuscript. Additionally, we include studies on the radiation patterns,

focusing on how the chemical potential and relaxation time of graphene influence the direction of the antenna's main beam.

In Supplementary Fig. 9, the total efficiency comparison between the simulated graphene antennas and the metal antenna is depicted.

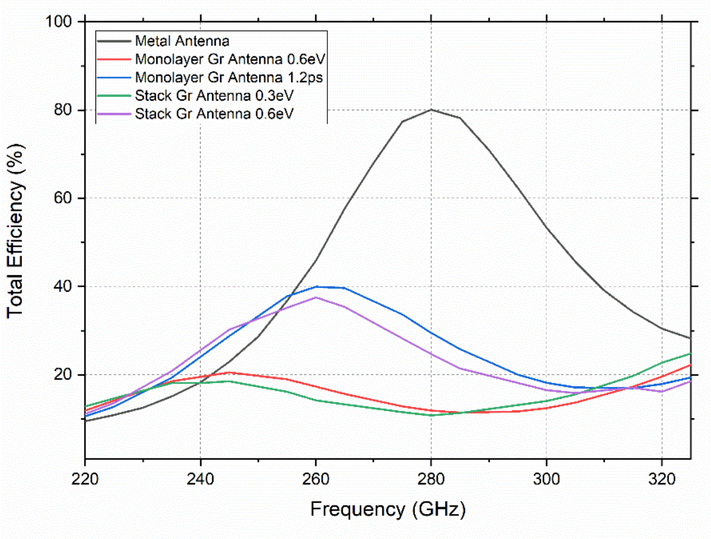

Supplementary Figure 9: Efficiency plot comparison of the simulated graphene antennas versus the metal antenna. The relaxation time of graphene is 1.2 ps.

The total efficiency of a metal antenna can reach up to 80% at the resonance, while graphene antennas tend to be much less efficient. For instance, a standard monolayer graphene antenna with a chemical potential of 1.2 eV and a relaxation time of 1.2 ps, which indicates very high conductivity, achieves only 40% total efficiency at resonance. In contrast, a graphene stack antenna, which features top and bottom graphene patches with half the chemical potential, can achieve efficiency values that are quite close to those of the monolayer configuration. This means that the graphene stack design can somewhat reduce the necessity for extremely high conductivity in graphene antennas.

The normalized realized gain plots in the H-plane and E-plane for the simulated antennas of Fig.6 (b) of the main manuscript text are presented in Supplementary Fig. 10.

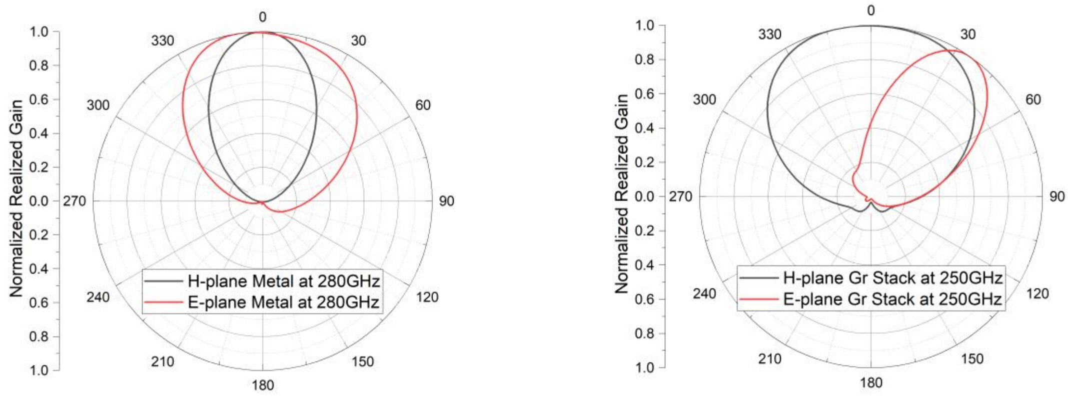

Supplementary Fig. 10: Simulated normalized gain in E-plane and H-plane of the metal and graphene stack antenna at the resonance frequency. Left: Metal Antenna. Right: Graphene Stack Antenna.

In addition to our simulation studies, we also examined the primary radiation direction in both the H-plane and the E-plane for graphene stack antennas with very low values of chemical potential and relaxation time. That is, we kept the relaxation time of graphene contact at 1.2 ps and varied the chemical potential between 0.01, 0.1, 0.2, and 0.3 eV. Additionally, we kept the chemical potential at 1.2 eV and varied the relaxation time of the graphene stack antenna between 0.01, 0.1, 0.2, and 0.3 ps. The normalized realized gain plots at antenna resonance in all cases are depicted in Supplementary Fig. 11.

The results show a tilt of the main beam in the E-plane for different graphene conductivities. For a fixed 1.2 ps of relaxation time and varying chemical potential, the real and imaginary parts of graphene impedance are changed, and the main beam direction goes from 34° for the graphene with the lowest conductivity to -18° for graphene with the highest conductivity. For a fixed 1.2 eV of chemical potential and varying relaxation time, the real part of graphene impedance is changed while the imaginary part is kept constant, and the main beam direction goes from 30° for the graphene with the lowest conductivity to 26° for the graphene with the highest conductivity.

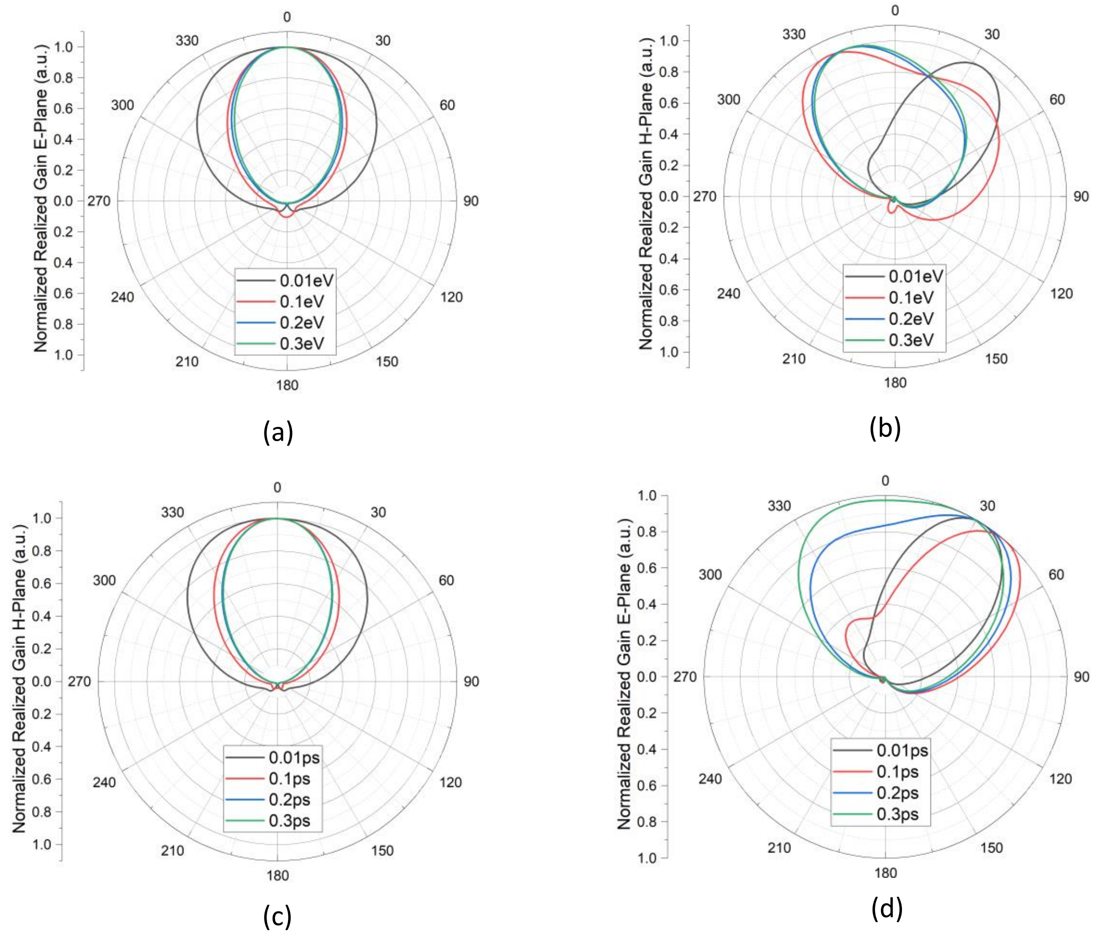

Supplementary Fig. 11: Simulated normalized realized gain of (a) graphene stack antennas with 1.2ps relaxation time and varying chemical potential in H-plane, (b) graphene stack antennas with 1.2ps relaxation time and varying chemical potential in E-plane, (c) graphene stack antennas with 1.2 eV chemical potential and varying relaxation time in H-plane, and (d) graphene stack antennas with 1.2 eV chemical potential and varying relaxation time in E-plane

REFERENCES

[1] V. P. Gusynin, S. G. Sharapov, and J. P. Carbotte, AC Conductivity of Graphene: from the Tight-Binding Model to 2+1-Dimensional Quantum Electrodynamics, *International Journal of Modern Physics B*, 2007, DOI: 10.1142/S0217979207038022

[2] V. P. Gusynin, S. G. Sharapov, and J. P. Carbotte, Magneto-Optical Conductivity in Graphene, *J. Phys.: Condens. Matter*, 2006, DOI: 10.1088/0953-8984/19/2/026222

[3] V. P. Gusynin, S. G. Sharapov, and J. P. Carbotte, Sum Rules for the Optical and Hall Conductivity in Graphene, *Phys. Rev. B*, 2007, DOI: 10.1103/PhysRevB.75.165407
